# Use of online clinical videos for clinical skills training for medical students: benefits and challenges

**DOI:** 10.1186/1472-6920-14-56

**Published:** 2014-03-21

**Authors:** Hye Won Jang, Kyong-Jee Kim

**Affiliations:** 1 Department of Social and Preventive Medicine, Sungkyunkwan University School of Medicine, 300 Cheoncheon-dong, Jangan-gu, Suwon, Gyeonggi-do 440-746, South Korea; 2 Department of Medical Education, Dongguk University School of Medicine, 32 Dongguk-ro, Ilsandong-gu, Goyang-si, Gyeonggi-do 410-820, South Korea

## Abstract

**Background:**

Multimedia learning has been shown effective in clinical skills training. Yet, use of technology presents both opportunities and challenges to learners. The present study investigated student use and perceptions of online clinical videos for learning clinical skills and in preparing for OSCE (Objective Structured Clinical Examination). This study aims to inform us how to make more effective us of these resources.

**Methods:**

A mixed-methods study was conducted for this study. A 30-items questionnaire was administered to investigate student use and perceptions of OSCE videos. Year 3 and 4 students from 34 Korean medical schools who had access to OSCE videos participated in the online survey. Additionally, a semi-structured interview of a group of Year 3 medical students was conducted for an in-depth understanding of student experience with OSCE videos.

**Results:**

411 students from 31 medical schools returned the questionnaires; a majority of them found OSCE videos effective for their learning of clinical skills and in preparing for OSCE. The number of OSCE videos that the students viewed was moderately associated with their self-efficacy and preparedness for OSCE (p < 0.05). One-thirds of those surveyed accessed the video clips using mobile devices; they agreed more with the statement that it was convenient to access the video clips than their peers who accessed the videos using computers (p < 0.05). Still, students reported lack of integration into the curriculum and lack of interaction as barriers to more effective use of OSCE videos.

**Conclusions:**

The present study confirms the overall positive impact of OSCE videos on student learning of clinical skills. Having faculty integrate these learning resources into their teaching, integrating interactive tools into this e-learning environment to foster interactions, and using mobile devices for convenient access are recommended to help students make more effective use of these resources.

## Background

Having students acquire competency in basic clinical skills is an important goal of medical education. As such, medical schools offer OSCE (Objective Structured Clinical Examination) to evaluate students for their clinical skills and they spend a significant amount of time self-studying clinical skills [1]. Therefore, it is important that medical schools offer students learning resources to support their self-study of clinical skills.

There has been a growing emphasis on improving the teaching and learning of clinical skills in Korean medical education as OSCE has become part of the national licensing examination since 2009. There have been challenges in medical schools to reform their curriculum on clinical skills as it requires a great deal of resources. As a response to this issue, e-learning has been adopted in Korean medical schools. The present study aims to investigate student experiences of using e-learning to learn clinical skills and to identify areas for improvement to advance the theory and practice of e-learning for clinical education.

Research shows that e-learning is effective in supporting clinical education. People learn effectively from multimedia instructions, and they are of particular importance for medical education [2]. Furthermore, educational videos afford us “to capitalize on the ability of moving images to teach procedures requiring skilled techniques and specialized physical examination [3].” Accordingly, video demonstrations of clinical skills have shown to improve learning of clinical skills [4-10] and medical students appreciate the availability of such learning resources [11,12].

Various formats are available for e-learning in clinical education. Among them, offering online videos on clinical skills (i.e., OSCE videos) is a popular format. Although the effectiveness of OSCE videos in learning outcomes are known, there is lack of research on how to make more effective use of them. Furthermore, there is little guidance on how to integrate e-learning into the curriculum despite the recommendation that information technology resources be integral part to supporting the clinical skills curriculum [13]. Use of technology presents both opportunities and challenges to learners [14]. Therefore, the present study investigated student experiences of the use of OSCE videos to identify benefits and challenges of e-learning in clinical skills training. In doing so, this study aims to inform the practice and theory to make more effective use of these resources.

In terms of the benefits of OSCE videos on student learning, the present study investigated the impact of using OSCE videos on factors that are known to be associated with student performance in OSCE. The literature suggests that self-efficacy, anxiety, perceived level of preparedness for OSCE can predict the student’s performance in OSCE [15,16]. Thus, the present study investigated the association between the number of OSCE video clips students viewed and their self-efficacy, anxiety, and preparedness for OSCE. Additionally, the present study investigated the use of mobile devices for using OSCE videos. With the development of information and communication technologies, the use of mobile devices in medical education is becoming increasingly popular [14,17]. Yet, research is still scant on how students perceive the mobile learning environment. Therefore, the present study examined student experiences of the mobile learning environment in using OSCE videos.

**Figure 1:**
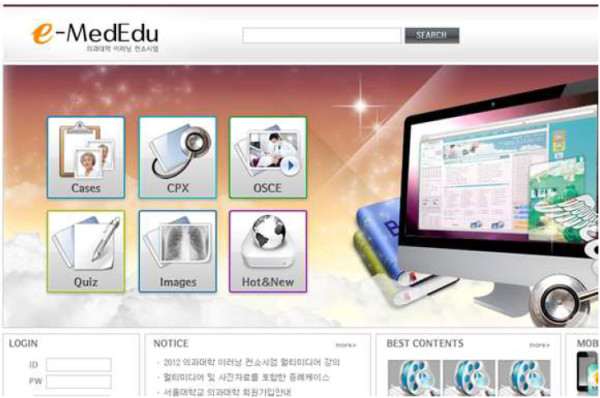
**e-MedEdu home page (**http://www.mededu.or.kr**).**

In the present study, students had access to OSCE videos offered by the Korean Consortium for e-Learning in Medical Education. This organization was formed to develop peer-reviewed online learning resources for medical students and launched an e-learning portal named e-MedEdu (http://www.mededu.or.kr) (see Figure 1). This website offers various types of learning resources, including approximately 300 video clips demonstrating basic clinical skills, such as clinical procedures and physical examination skills. These video clips include narrations and captions for instructions and are usually 10–20 minutes in length. These video clips are streamed live. More detailed information about e-MedEdu is provided elsewhere [18]. This e-learning portal has also been available in mobile applications on both Android and iPhone platforms since Spring, 2011.

## Methods

A mixed methods study was conducted for this evaluation study. First, we developed a self-report questionnaire to investigate student use and perceptions of OSCE videos, which encompasses 30 items. The first part of the questionnaire consists of 15 items regarding the participants’ demographics and how they used OSCE videos to learn clinical skills. The second part of the questionnaire comprises ten items which ask the participants to indicate their attitudes toward OSCE that are known to be associated with their performance in OSCE – i.e., self-efficacy, test anxiety, and preparedness for OSCE. These items were adopted from questionnaires reported in the relevant literature [15,16]. The last part of the questionnaire comprises five items regarding the participants’ perception of the effectiveness of OSCE videos. These last five items were constructed and reviewed by the authors, and a reliability analysis was performed on these items to evaluate their internal consistency. The Chronbach’s alpha was 0.88, which was regarded as an acceptable level [19]. Participants rated their responses on a four point Likert scale ranging from 1 (strongly disagree) to 4 (strongly agree) except for one item regarding respondent confidence in his or her overall clinical skills, which was on a 6-point Likert scale, where 1 = “little or no confidence” and 6 = “very high confidence”.

Year 3 and 4 students from 34 medical schools registered in the e-learning portal mentioned above (approximately 3,000 students) were invited to participate in the survey by sending them an e-mail with a link to the online survey site. Participants took part in the survey by agreeing to the consent form before they began the survey. The questionnaire was self-administered and answered anonymously. Year 1 and 2 students, which are pre-clinical years, were excluded from this study as clinical skills are taught during clinical years in most Korean medical schools [20].

Second, an interview study was conducted for an in-depth understanding of student experiences with the OSCE videos. Four 3^rd^ year students from Sungkyunkwan Medical School in Seoul, Korea were interviewed as a group for 45 minutes using a semi-structured interview method. The authors facilitated the interview session. Additional qualitative data were collected from written responses to an open-ended question in the questionnaire conducted earlier. Additionally, log data was retrieved from the server and was analyzed for usage statistics on OSCE videos.

Descriptive statistics were used to analyze frequencies of responses on quantitative data. Student *t*-test and one-way ANOVA were conducted for comparison of responses between or among groups. Chi-squared test was performed for nonparametric tests of statistical significance. All significance was evaluated at a confidence level of 95%. All statistical analyses were conducted using SPSS version 18. For qualitative data, the interview was transcribed and analyzed by the authors using a thematic analysis method to identify emerging themes.

Data were collected throughout the academic year 2011–2012. IRB approval was not requested, because this study fell under the general exemption from our institutional review board for educational outcomes data.

## Results

### Participant demographics and their use of OSCE videos

411 students from 31 medical schools across the nation completed and returned the questionnaire. Among these participants, 47% were in Year 3 and 53% were in Year 4. 27% of the total respondents were female and 73% were male; this is in line with the gender ratio of the population, which is approximately 30% female and 70% male. Participant ages ranged from 23 to 39 years (M = 27.5, SD = 3.8). In terms of the use of mobile devices, 87% of the total participants reported that they owned a mobile device (i.e., smart phones or tablets).

**Figure 2:**
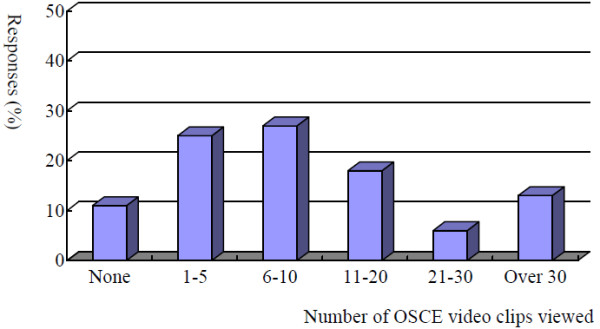
**Number of OSCE video clips participants viewed during the academic year (*n*** **= 411).** Number of OSCE video clips viewed. Responses (%)

Figure 2 shows the number of OSCE video clips participants viewed during the academic year. Having participants divided into three age groups (1 = 25 years or younger, 2 = between 26 and 30, 3 = over 30), there were no differences in the distributions of the number of OSCE videos viewed across all the age groups (*χ*^2^ = 8.12, df = 10, *p* = 0.62). Also, no differences were observed in gender ratios in the number of OSCE videos participants viewed (*χ*^2^ = 8.45, df = 5, *p* = 0.13). Additionally, having participants divided into three groups according to their confidence levels on their overall clinical skills (1–2 = low, 3–4 = medium, 5–6 = high), the number of OSCE videos viewed were comparable across these three groups (*χ*^2^ = 14.85, df = 10, *p* = 0.14).

Table 1 shows participant responses on reasons for using OSCE videos. 42% of those surveyed answered that they used the video clips to review clinical skills that were difficult to understand just by taking lectures. 37% responded that they used these clips to refresh their memory before they practiced the procedures. Additionally, 13 answered that they used the video clips to learn clinical skills that they could not observe directly in classes or during clerkships. 8% answered that they watched the videos clips to learn skills on their own that were not taught in classes.

**Table 1:** Participant responses on reasons for using OSCE videos

**Items**	**Response (%)**
To review clinical skills difficult to understand by just taking lectures	173 (42%)
To refresh my memory before I practice procedures	152 (37%)
To learn skills that could not be observed directly	53 (13%)
To learn skills that were not taught in classes	33 (8%)
Total	411 (100%)

The log data was analyzed to identify types of OSCE videos students accessed the most. Four out of the top five OSCE videos with the most hits were on physical examinations – i.e., neurological exanimations (6,800 hits), respiratory examination (6,700 hits), cardiac exanimation (5,700 hits), and otoscopic examination (4,800 hits). The video clip on applying splints was the fourth among the OSCE videos with the most hits (4,800 hits).

### Participant perceptions of the effectiveness of OSCE videos

**Figure 3:**
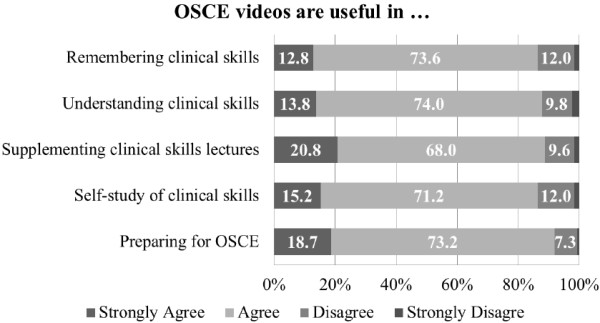
**Participant perceptions of effectiveness of OSCE videos (*n*** **= 411).**

A majority of participants perceived that OSCE videos were useful in learning clinical skills and in preparing for OSCE (see Figure 3). Participants’ perceived effectiveness of OSCE videos were comparable among groups of different levels of self-efficacy on OSCE (*F* = 30.77, df = 20, *p* = 0.60) and also across years of study (*F* = 6.80, df = 10, *p* = 0.60).

Table 2 shows correlations between the number of OSCE videos participants viewed and their attitudes towards OSCE. Participant perceptions of their self-efficacy and preparedness for OSCE were significantly positively correlated with the number of OSCE videos they viewed (*p* < 0.05). No correlation was found between the participants’ anxiety of OSCE and the number of OSCE videos they viewed (*p* = 0.24).

**Table 2:** Correlations between the number of OSCE video clips viewed and variables associated with student performance in OSCE (correlation coefficients)

**Variables**	**1**	**2**	**3**
1. # of video clips viewed	-		
2. Self-efficacy	0.19*	-	
3. Anxiety	- 0.24	- 0.17	-
4. Preparedness for OSCE	0.22*	0.56**	- 0.22

*p < 0.05, **p < 0.01.

Both survey respondents and interview participants pointed out the rich learning resource as the benefit of using video clips for learning clinical skills. Before introducing them to OSCE videos, the main resource for the students’ learning of clinical skills was textbooks. As one student mentioned “OSCE videos helped me learn clinical skills better by watching demonstrations than by just reading textbooks. I used OSCE videos when there was something unclear about the instructions on clinical procedures written in the textbook. That’s much better than just reading texts.” Another student noted that “it is often hard for me to watch a professor demonstrate a clinical procedure from where I am sitting in the classroom. I can observe the procedures more clearly in video demonstrations.”

Additionally, several survey respondents appreciated the efforts made by the consortium to publish quality instructional videos for them. One student wrote “I appreciate the efforts by the consortium to offer such useful learning resources for us. They helped me prepare for OSCE a lot.”

### Using mobile devices to access OSCE videos

The OSCE videos that participants in the present study used are also accessible on mobile devices. The OSCE video clips received approximately 17,000 hits per month on average in year 2012, and 37% of them were from mobile devices. 38% of those surveyed (*n* = 156) reported that they used mobile devices as a primary device for accessing the OSCE videos.

All of those answered that they were using mobile devices to access the OSCE videos agreed or strongly agreed with the statement that mobile applications helped them access the videos more conveniently and they agreed more with the statement that it was more convenient to accessed the video clips through mobile devices compared with their peers who accessed them using computers (*t* = 7.74, *p* < 0.01).

### Barriers to using OSCE videos

Some participants in this study mentioned the lack of interactions as a challenge to their learning using OSCE videos. One student stated “I had some questions while I was viewing the video clips. I hoped there was an online communication tool on the website so that I could post questions and get answers from professors or other students.” Students also pointed out the lack of integration into curriculum as a barrier to using OSCE videos. As one student commented, “I think these video clips are very good, but I think only a few professors are aware of these videos. I hope professors use these videos when they teach us. I think that will help us be more aware that the video clips are available.”

Both qualitative and quantitative data from the present study show that participants experienced technical difficulties in accessing video clips on mobile devices in the early stage of its implementation. Some interview participants pointed out the problem of low connection speed in some video clips in mobile settings. Survey data also revealed that those who accessed the videos on mobile devices more likely disagreed with the statement that the connection speed was good compared with their peers who acceded them using computers (*t* = 3.17, *p* < 0.01).

## Discussion and conclusions

In line with other previous studies, the present study shows that students have positive perceptions of OSCE videos and that they make use of these videos in various ways to support their self-study of clinical skills. Our findings indicate that e-learning can be a fruitful venture in improving clinical education by meeting the needs of students to supplement traditional teaching of clinical skills. Still, some students have negative responses on the effectiveness of OSCE videos, as shown in Figure 3. It can be speculated that watching video clips alone is not enough for some students to learn clinical skills. This is also in line with our findings that OSCE videos need to be integrated into faculty teaching to make more effective use of them.

The present study indicates a successful implementation of collaboration of Korean medical schools in using e-learning to support clinical skills training. Despite the benefits of e-learning in clinical education, the major barrier to implementing it is the lack of resources in medical schools. The key to overcoming these challenges in this study was collaboration among medical schools. Collaboration is a key paradigm for innovating medical education [21]. As medical schools often face challenges in implementing OSCE with limited resources, sharing resources is an effective and efficient way to support student learning of clinical skills. This particularly would have great value for students in medical schools in resource-constrained environments.

Our findings have implications for improving the practice of using OSCE videos for the teaching and learning of clinical skills. First, the present study shows that faculty makes little use of OSCE videos in their teaching. This finding indicates that e-learning has made little impact on faculty’s teaching practice despite of the popular usage by students. Faculty development is needed on how to integrate OSCE videos into their teaching. For instance, professors can have students watch some video clips before they come to class to have them prepared for the class, thus reducing time for lectures and giving more time for practice and feedback. To that end, it is recommended that best practices on making use of OSCE videos for teaching clinical skills be explored and disseminated. Second, our findings show that students may face challenges in learning with OSCE videos without interacting with others. As educational videos are being published in peer-reviewed journals and live streaming of surgical demonstrations are gaining popularity, there are increasing efforts to integrate interactive tools – i.e., social networking services - into such environments [9]. Future research is recommended to inform the theory and practice for the effective use of such interactive tools.

Our study indicates that the mobile learning environment benefits medical students by offering convenient access to OSCE videos. As students spend a significant amount of time outside the classroom during clinical rotations, providing convenient access to learning resources may bring particular benefits for them by helping create a seamless learning environment, in which learning can take place across time and locations using various platforms [22]. Nonetheless, students reported the problem of low connection speed in mobile settings, which was caused by the format of some video clips that was not optimized for the mobile environment in the early stage of our study. These problems were resolved by optimizing the video format for wireless mobile connections. Therefore, it is suggested that streaming videos be optimized for the wireless network connection setting and tested before implementation to prevent connection speed problems.

The present study has some limitations. First, the low response rate of the survey has the potential to cause bias in the results of the study. We made attempts to increase the response rate by sending a reminder to participants the following week and having those who completed the survey eligible for prizes. Furthermore, efforts were made to triangulate the data sources by conducting both a survey study and an interview study. Second, the impact of OSCE videos on student performance in OSCE was studied using correlation analysis only. Future study is recommended of factors associated with student performance in OSCE using a multivariate analysis for a better understanding of factors that influence student performance in OSCE.

## Competing interests

No competing interests exist in this study.

## Authors' contributions

HJ contributed to acquisition, analysis, and interpretation of data. KK contributed to conception, design, and acquisition of data. Both authors critically reviewed the manuscript for revision and approved the version to be publisihed.

## Pre-publication history

The pre-publication history for this paper can be accessed here:

http://www.biomedcentral.com/1472-6920/14/56/prepub
